# Intrinsically Self-renewing Neuroprogenitors From the A/J Mouse Spiral Ganglion as Virtually Unlimited Source of Mature Auditory Neurons

**DOI:** 10.3389/fncel.2020.599152

**Published:** 2020-12-09

**Authors:** Francis Rousset, Vivianne B. C. Kokje, Rebecca Sipione, Dominik Schmidbauer, German Nacher-Soler, Sten Ilmjärv, Marta Coelho, Stefan Fink, François Voruz, Antoun El Chemaly, Antoine Marteyn, Hubert Löwenheim, Karl-Heinz Krause, Marcus Müller, Rudolf Glückert, Pascal Senn

**Affiliations:** ^1^The Inner Ear and Olfaction Lab, Department of Pathology and Immunology, Faculty of Medicine, University of Geneva, Geneva, Switzerland; ^2^Department of Clinical Neurosciences, Service of ORL & Head and Neck Surgery, University Hospital of Geneva, Geneva, Switzerland; ^3^Department of Otolaryngology, Medical University of Innsbruck, Innsbruck, Austria; ^4^Department of Pathology and Immunology, Faculty of Medicine, University of Geneva, Geneva, Switzerland; ^5^Department of Otorhinolaryngology-Head and Neck Surgery, University of Tübingen, Tübingen, Germany

**Keywords:** cochlea, auditory neuron, regeneration, otic neurospheres, organoids, 3R, reduce

## Abstract

Nearly 460 million individuals are affected by sensorineural hearing loss (SNHL), one of the most common human sensory disorders. In mammals, hearing loss is permanent due to the lack of efficient regenerative capacity of the sensory epithelia and spiral ganglion neurons (SGN). Sphere-forming progenitor cells can be isolated from the mammalian inner ear and give rise to inner ear specific cell types *in vitro*. However, the self-renewing capacities of auditory progenitor cells from the sensory and neuronal compartment are limited to few passages, even after adding powerful growth factor cocktails. Here, we provide phenotypical and functional characterization of a new pool of auditory progenitors as sustainable source for sphere-derived auditory neurons. The so-called phoenix auditory neuroprogenitors, isolated from the A/J mouse spiral ganglion, exhibit robust intrinsic self-renewal properties beyond 40 passages. At any passage or freezing–thawing cycle, phoenix spheres can be efficiently differentiated into mature spiral ganglion cells by withdrawing growth factors. The differentiated cells express both neuronal and glial cell phenotypic markers and exhibit similar functional properties as mouse spiral ganglion primary explants and human sphere-derived spiral ganglion cells. In contrast to other rodent models aiming at sustained production of auditory neurons, no genetic transformation of the progenitors is needed. Phoenix spheres therefore represent an interesting starting point to further investigate self-renewal in the mammalian inner ear, which is still far from any clinical application. In the meantime, phoenix spheres already offer an unlimited source of mammalian auditory neurons for high-throughput screens while substantially reducing the numbers of animals needed.

## Introduction

Nearly 460 million individuals are affected by sensorineural hearing loss (SNHL), one of the most common human sensory disorders (Olusanya et al., 2019). Because of the lack of regenerative capacity of the sensory epithelia and spiral ganglion neurons (SGNs), SNHL is permanent. Studies aiming at otoprotection and regeneration were typically focusing on the sensory epithelium as a primary target. In the recent past, the SGNs have been more intensively studied, and damage of auditory neurons has been reported in multiple common conditions including noise-induced hearing loss (Kujawa and Liberman, 2009), presbycusis (Moser and Starr, 2016), head trauma (Vartiainen et al., 1985), or endolymphatic hydrops (Bixenstine et al., 2008).

The auditory synapse is affected in early SNHL, with a decreased number of synaptic ribbons between inner hair cells (IHCs) and auditory neurons leading to diminished sound discrimination without detectable increase in hearing thresholds (Kujawa and Liberman, 2009). This so-called hidden hearing loss can lead to tinnitus and usually precedes the complete degeneration of auditory neurons (Xiong et al., 2019). In case of SNHL, the IHCs can be bypassed by cochlear implants, which directly stimulate the SGNs. As opposed to IHC loss, the loss of SGNs cannot be compensated in humans (Michelson et al., 1973).

Regeneration of the sensory epithelium occurs naturally in lower vertebrates through proliferation and transdifferentiation of the supporting cells (Corwin and Cotanche, 1988; Ryals and Rubel, 1988). In this process, the realignment of the auditory synapse between the newly formed IHC and auditory neurons is essential and relies on the high plasticity of auditory neurons. In contrast, the mature mammalian cochlea lacks the capacity for efficient regeneration (Warchol et al., 1993). Inner ear progenitor cells are present in mammals; however, their number rapidly decreases with age (Oshima et al., 2007; Moon et al., 2018).

It has been described that stem and progenitor cells can be isolated from the inner ear and cultured as neurospheres (Oshima et al., 2009). *In vitro*, self-renewal has been reported for neurospheres derived from neonatal mouse and human fetal spiral ganglion cells (SGCs; Senn et al., 2007; Roccio et al., 2018). Previous studies have identified the characteristics of differentiation, neurite outgrowth, and their electrophysiological activity (Li et al., 2016). *In vitro*, the newly formed mature auditory neurons respond to glutamate, firing action potentials and exhibiting similar features as auditory neuron explants (Martinez-Monedero et al., 2008; Chen et al., 2012). Auditory progenitor neurospheres are therefore a useful tool for explorative studies aimed at development of cellular therapies and improvement of the efficiency of cochlear implantation.

Previous studies have been able to use the neurospheres for at least five to seven passages, but they generally rapidly lose their self-renewal abilities and reach senescence, limiting their use (Oshima et al., 2007; Senn et al., 2007). The reason of this early sentence is still poorly understood, and overcoming this barrier could have major consequences for the development of future cell therapies.

In this study, we provide phenotypical and functional characterization of a novel robust model for auditory progenitors as sustainable source of sphere-derived SGCs *in vitro*. The so-called phoenix auditory neuroprogenitors, isolated from the A/J mouse cochlea, exhibit robust self-renewal properties (up to 40 passages; >10^12^-fold theoretical amplification), whereas in the same culture conditions, neurospheres from C57Bl6 neonatal mice were senescent after three passages. At any passage, phoenix spheres could be easily frozen, thawed, and reliably differentiated into mature auditory neurons and glial cells, expressing their phenotypic markers and exhibiting similar functional properties as native auditory neurons and human sphere-derived auditory neurons. This new model overcomes the limits of the previously described neurospheres, allows high-throughput screening assays, and represents a unique tool to understand auditory neuroprogenitor self-renewal and regeneration in mammals. To the best of our knowledge, this is the first primary culture model in the auditory field that may greatly reduce animal numbers with major 3R implications.

## Materials and Methods

### Study Approval

All animal procedures were approved by the local veterinary office and the Commission for Animal Experimentation of the Canton of Geneva, Switzerland, authorization number GE/189/17. Procedures using human fetal material were performed with full approval by the Ethics Committee of the Geneva State, Switzerland, and following signed informed consent of the donors.

### SGCs Isolation and *In vitro* Culture

Collection of mouse and human fetal inner ear SGCs was done as previously described in (Oshima et al., 2009; Roccio et al., 2018). P2–P5 A.B6-*Tyr*^+^/J [stock no. 002565, Jackson; carrying the wild-type allele of *Tyr* in an A/J strain genetic background (stock no. 000646)] and C57Bl6/J (stock no. 000664) pups were used. They were euthanized by decapitation. The head surface was sterilized with 70% ethanol and was sectioned along the midsagittal plane. Brain and brainstem were removed, and both half-skulls were immersed in 2.5 ml of ice-cold Hanks balanced salt solution (HBSS) in a 35-mm Petri dish. The cochleae were removed under a stereo microscope (Nikon, Japan). The cartilaginous cochlear capsule was opened, and the stria vascularis and the organ of Corti were removed in one piece from base to apex using 5.5 forceps. The modiolus (with the SGCs) was then transferred into 1.5-ml Eppendorf tube with ice-cold HBSS. For human fetal samples, inner ears were isolated from aborted human fetuses ranging from week 8 (W8) to W12 after conception. The postmenstrual date was used for the calculation of the fetal stage. For both human and mouse samples, tissue dissociation was done by enzymatic digestion followed by mechanical trituration. The modiolus was treated with Accutase (StemPro™ Accutase™ Cell Dissociation Reagent) for 15 min; the reaction was stopped by adding proliferation media: Dulbecco’s Modified Eagle Medium (DMEM):F12 with 15 mM HEPES buffer and 2 mM L-glutamine supplemented with 1× N2 and B27 supplement (Thermo Fisher Scientific) and 1× penicillin streptomycin (100 U/ml; Thermo Fisher Scientific), in presence of basic fibroblast growth factor (bFGF; 10 ng/ml, ProSpec), Insulin like growth factor 1 (IGF1; 50 ng/ml, Cell Guidance Systems), heparan sulfate (50 ng/ml, Sigma–Aldrich), and epidermal growth factor (EGF; 20 ng/ml, Cell Guidance Systems), and mechanical trituration was performed with P1000 (10–15 up and downs). Following Accutase removal with a centrifugation step, the cell suspension was filtered with a 70-μm filter to eliminate undissociated tissue or bony parts and select small cluster of SGCs. Isolated cells were maintained in culture with proliferation media in ultralow-attachment six-well plates (Corning, Sigma–Aldrich). Passaging of SGC-derived neurospheres was done twice a week using enzymatic digestion with Accutase™ followed by mechanical dissociation as previously described (Oshima et al., 2009). Differentiation was performed following passaging by seeding 200,000 or 32,000 isolated neuroprogenitors cells, respectively, in a 6- or 96-well plate coated with Matrigel (1:100 dilution, hESC qualified, Corning, Sigma–Aldrich). Differentiation was carried out after withdrawal of mitogenic growth factors in presence of differentiation medium (DMEM/F12, N2, B27, penicillin streptomycin, LIF 100 μg/ml, NT3 10 μg/ml, BDNF 10 μg/ml) for 1–7 days as specified in the figure legend for every experiment. The differentiation medium was changed twice a week. For all experiments, phoenix auditory cells were used between passage 15 and 30, unless stated otherwise in the figure legend. The relatively late passage usage allowed substantial material amplification, conservation through freezing and shipping of the cells for abroad experiments. Three different sources of phoenix cells were used in the study.

### Cell Counting

Following sphere/tissue dissociation with Accutase, neuroprogenitors were counted manually using a FAST READ 102^®^ (Biosigma) hemocytometer, according to the manufacturer’s instructions. Cell number was determined for three independent cultures up to passage 10, allowing the calculation of generation time (*G* = 5, 697 days) using the formula LOG10(2)/average slope (0.0528 ± 0.00165) and the theoretical global amplification after 40 passages (corresponding to 42.125 generations): 2^∧^42.125 = 4,79513E + 12-fold.

### Cell Cycle Analysis by Flow Cytometry

The cell cycle of phoenix auditory neurospheres was studied by propidium iodide staining of the DNA followed by fluorescence activated cell sorting analysis. Following dissociation, 1 × 10^5^ to 2 × 10^5^ cells were centrifuged, fixed with a 70% ice-cold ethanol solution, treated with RNase A, and then stained with the DNA-binding agent propidium iodide (Sigma–Aldrich) in phosphate-buffered saline (PBS) buffer overnight at 4°C. The cell sorting analysis was performed on a BD Accuri C6. FlowJo (v. 10.6.2) software was used to determine the percentage of cells engaged in the cell cycle (proliferating).

### Measurement of Ca^2+^ Cytosolic Release Induced by Glutamate

A total of 32,000 cells/well were differentiated to auditory neurons on a 96-well plate coated with Matrigel, as previously described (Rousset et al., 2020). Differentiated cells were loaded with FLUO-8 (Interchim), according to the manufacturer’s protocol. After an incubation period of 45 min at 37°C, the glutamate-induced cytosolic calcium release was assessed in a FDSS/μCELL Functional Drug Screening System (Hamamatsu). The neuronal kinetics of calcium release was followed over 10 min following glutamate addition, with one measure every 0.5 s.

### Video Time-Lapse Microscopy

Seven days’ differentiated phoenix cells, human auditory SGNs, or C57Bl6 mouse explants were loaded with FLUO-8 (Interchim) according to the manufacturer’s protocol. After an incubation period of 45 min at 37°C, samples were stimulated with 100 μM glutamate, kainate, or ATP. Because of the low number of human fetal samples (*n* = 3 for the whole study), human sphere-derived auditory neurons were first stimulated with kainate (as in Figure 7K). Given the absence of response, medium was removed, and cells were stimulated with ATP a few minutes later. Therefore, pictures on Figures 7J,K were recorded on the same sample. Fluorescence kinetics was recorded for 3 min with one picture per second and an acquisition time of 0.2 s per picture on a Zeiss Axio Observer Z1 with a Definite Focus 2 microscope.

**Figure 1:**
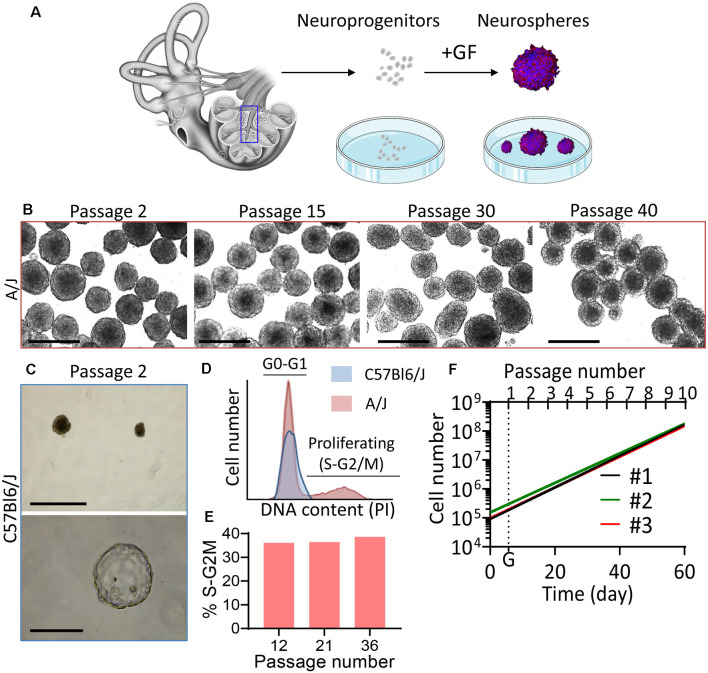
High intrinsic propagation potential of phoenix auditory neuroprogenitors. **(A)** Auditory neuroprogenitors were isolated from the mouse pup spiral ganglion and cultured as single cell suspension. Upon growth factor addition (FGF, EGF, IGF, heparan sulfate), auditory neuroprogenitors form neurospheres that can be propagated. **(B,C)** Bright-field microscopy pictures of spheres obtained from **(B)** A/J and **(C)** C57Bl6/J P5 mouse pups after 3 days in culture following the indicated passage. **(B)** Scale bar: 500 μm. Spheres from C57Bl6/J rapidly reach senescence (upper panel; scale bar: 500 μm) and some become translucent (hollow) spheres (lower panel; scale bar: 100 μm). **(D)** DNA content was quantified by flow cytometry in A/J (red) and C57Bl6/J (blue) auditory neuroprogenitors using propidium iodide (PI) staining. At the fourth passage, A/J neuroprogenitors exhibited more than 35% cells in S/G2-M phases, whereas C57Bl6 were quiescent (100% cells in G0–G1 phases). **(E)** The proportion of proliferating progenitors remained constant (>35%) over passages. Bar graph shows the relative proportion of phoenix neuroprogenitors engaged in S–G2M phases of the cell cycle at passage 12, 21, and 36. **(F)** The high proliferation rate of phoenix neuroprogenitors was confirmed with systematic counting of cells up to passage 10. #1, #2, and #3 stand for independent cultures from three different litters. G (dotted line) represents the average generation time for the three cultures and is equal to 5,697 days.

**Figure 2:**
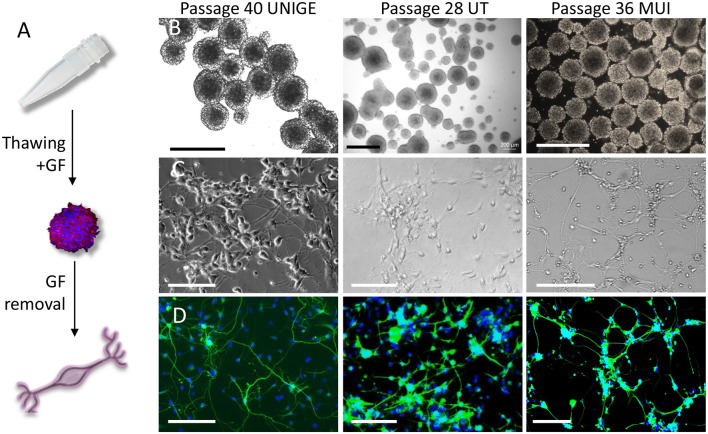
Propagation and differentiation of phoenix auditory neuroprogenitors following freezing/thawing cycles. **(A)** Upon thawing, progenitors rapidly form highly propagating auditory neurospheres, which can be easily differentiated into auditory neurons like cells with appropriate coating and growth factor removal. **(B)** Bright-field microscopy pictures of phoenix neuroprogenitors as cultured at the indicated passage in ultralow attachment flasks following freezing and thawing cycles in three different laboratories. Scale bar: 500 μm. **(C)** Bright-field pictures and **(D)** BIII-tubulin staining of differentiated auditory neurons on Matrigel. Scale bar: 100 μm. At any passage in each of the three institutions, phoenix neurospheres were differentiated into auditory neuronal cells exhibiting a majority of bipolar neurons. UNIGE, University of Geneva; UT, University of Tübingen; MUI, University of Innsbruck.

**Figure 3:**
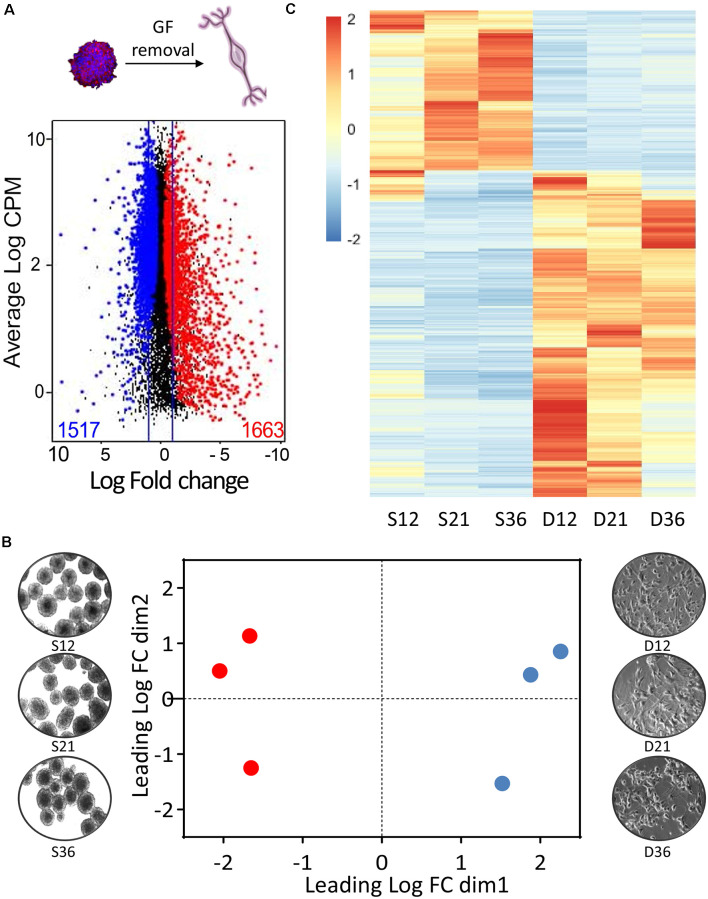
Transcriptomic profile of phoenix auditory neurons. **(A)** MD plots (mean difference plot of expression data) comparing gene expression in neurospheres and D7 differentiated neurons. Significantly differentially expressed genes at a false discovery rate (FDR) below 5% are highlighted in blue when enriched in neuroprogenitors and in red when enriched in auditory neurons. The lines represent the 2-fold expression change threshold. CPM (counts per million) refers to the number of reads of the gene of interest relatively to the total numbers of reads and is representative of the relative expression of the transcript within the dataset. **(B)** Multidimensional scanning plot based on the fold changes between all the pairs of samples. **(C)** Heatmap representing significantly differentially expressed genes between progenitors (S12, S21, S36; column 1–3) and differentiated neurons (D12, D21, and D36; column 4–6). S, spheres; D, differentiated. The number in the sample names corresponds to the passage number of the cells, which are from three independent sources.

**Figure 4:**
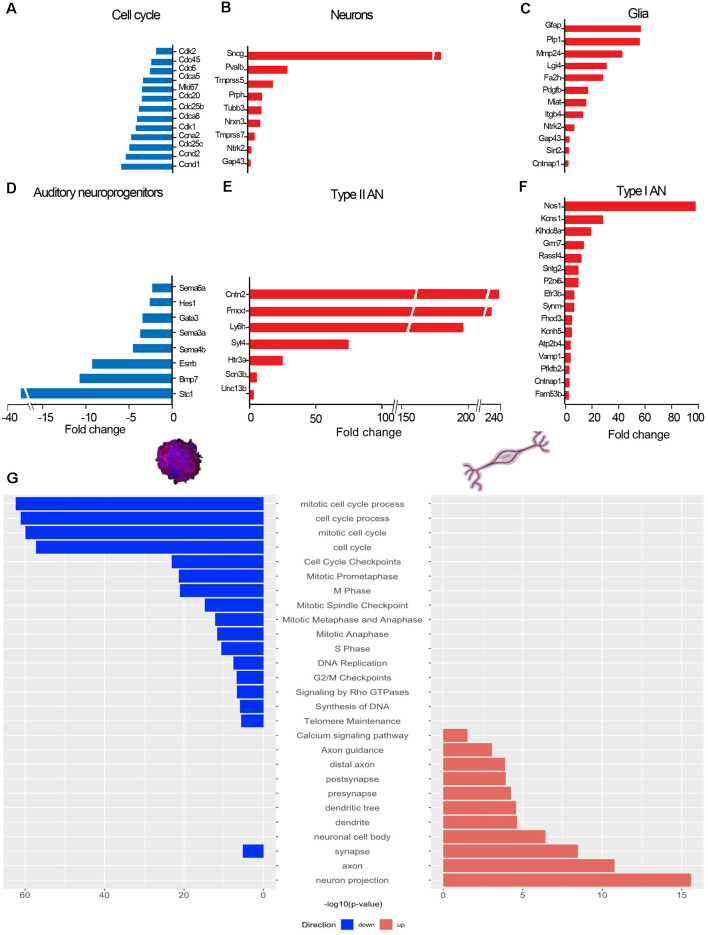
Changes in gene expression upon differentiation. RNAseq-based transcriptome analysis of phoenix auditory neurons (D7 differentiation). mRNA levels in differentiated cells are expressed relatively to neuroprogenitor spheres of equivalent passage (samples were paired at passage 12, 21, and 36 forming a triplicate). **(A–F)** Bar graphs showing changes in expression of main genes related to **(A)** cell cycle, **(B)** neural differentiation, **(C)** glial differentiation, **(D)** neuroprogenitor markers, **(E)** type II auditory neurons, and **(F)** type I auditory neuron in differentiated phoenix auditory neurons relatively to neuroprogenitors spheres. All genes showed in the bar graphs were significantly differentially expressed (FDR < 0.05). **(G)** Main relevant gene ontologies showing a significantly different level in mature neurons (red) vs. neuroprogenitors (blue). Results are representative of the average from triplicates of cells originating from three different litters.

**Figure 5:**
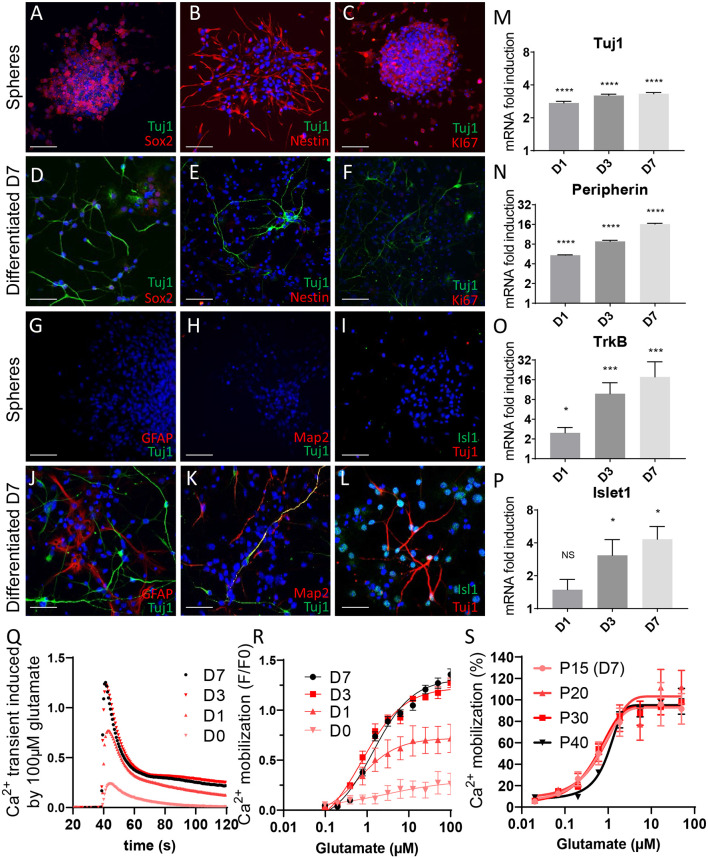
Phenotypical characterization of phoenix auditory neurons. **(A–F)** Three days following passaging, neurospheres were plated on Matrigel-coated coverslips. **(A–C)** Four hours following the plating, neuroprogenitor spheres were fixed, permeabilized, and stained for the indicated markers. **(D–F)** Seven days’ differentiated auditory neurons were used as control. **(A,D)** BIII-tubulin and Sox2 in neuroprogenitors and differentiated cells. **(B,E)** BIII-tubulin and nestin in neuroprogenitors and differentiated cells. **(C,F)** BIII-tubulin and KI67 in neuroprogenitor spheres and differentiated auditory neurons. Scale bar: 50 μm. Isolated phoenix auditory neuroprogenitors were differentiated on Matrigel-coated **(G–L)** coverslips for immunofluorescence experiments, **(M–P)** six-well plates for qPCR experiments, or **(Q–R)** 96-well plates for Ca^2+^ transients measurement for the indicated time points (1–7 days). **(G–L)** Seven days’ differentiated auditory neurons were fixed, permeabilized, and stained for the indicated markers. **(G–I)** Undifferentiated spheres of progenitors were used as control. **(G,J)** BIII-tubulin and astrocyte marker GFAP, **(H,K)** BIII-tubulin and mature neurons marker Map2, **(I,L)** BIII-tubulin and sensory neurons marker Isl1, **(M–P)** mRNA level of several auditory neuron markers such as **(M)** BIII-tubulin, **(N)** peripherin, **(O)** TrkB, and **(P)** Islet1 was assessed by qPCR during the differentiation process (after 1, 3, or 7 days of differentiation). Scale bar: 50 μm. **(Q,R)** Phoenix auditory neurons were differentiated for the indicated times, loaded with Ca^2+^-sensitive ratiometric probe (FLUO-8), and stimulated with increasing concentrations of glutamate (0.02–100 μM). Traces represent **(Q)** maximum amplitudes (*F*/*F*_0_) in response to 100 μM glutamate and **(R)** dose–response curves, as recorded along the differentiation process at different time points (after 1, 3, or 7 days of differentiation). **(S)** Glutamate dose–response induced Ca^2+^ mobilization in D7 differentiated phoenix auditory neurons as recorded at different passages (from P15 to P40). Data represent the average ± SEM of three independent experiments. *p < 0.05, ***p < 0.005, ****p < 0.0005, NS: non significant.

**Figure 6:**
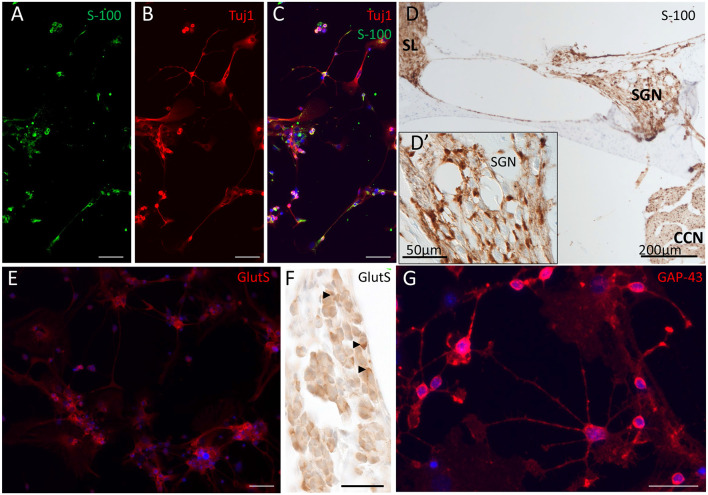
Glial marker expression in differentiated phoenix cells. **(A–C)** Beta-III-tubulin and S-100 staining 3 days after seeding of phoenix auditory neuroprogenitors. **(A)** S-100 (green). **(B)** Beta-III-tubulin (TUBB; red). **(C)** Composite image with DAPI nuclei staining. **(D)** S-100 staining in mouse pups cochlear slices. **(D′)** Inset represents a magnified view of the SGN area and identify Schwann cells as well as satellite glia cells positively stained. **(E,F)** Glutamine synthetase (GlutS) immunostaining shows presence in several cells mainly in very small satellite cells. **(F)** Glutamine synthetase in the spiral ganglion of a mouse, 1 day after birth (arrows). **(G)** GAP-43 immunostaining in differentiating (D3) phoenix cells. Scale bar for **(A,B,C,E)** = 50 μm. Scale bar for **(F)** and **(G)** = 40 μm.

**Figure 7:**
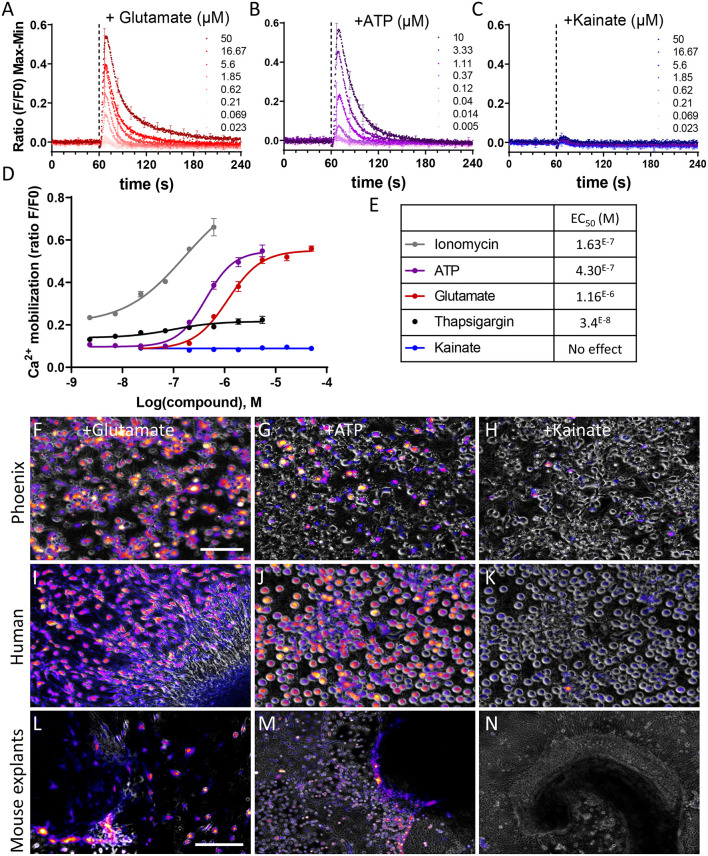
Phoenix and human fetal auditory neurons exhibit glutamatergic and purinergic Ca^2+^ transients. **(A–C)** Kinetics of Ca^2+^ transients induced by **(A)** glutamate, **(B)** ATP, **(C)** kainate in phoenix auditory neurons after 7 days of differentiation. Phoenix auditory neurons were loaded with Ca^2+^-sensitive ratiometric probe (FLUO-8) and stimulated with increasing concentrations of glutamate (0.02–50 μM), ATP (0.005–10 μM), or kainate (0.02–50 μM). **(D)** Dose–response curves were extracted from the max–min amplitude values, allowing the determination of an EC_50_ value **(E)** Ionomycin and thapsigargin were used as positive control. Data represent the average ± SEM of three independent experiments. **(F–N)** Live Ca^2+^ imaging of **(F–H)** phoenix auditory neurons, **(I–K)** human sphere-derived auditory neurons, and **(L–N)** mouse cochlear explants following stimulation with 100 μM glutamate **(F,I,L)**, ATP **(G,J,M)**, or kainate **(H,K,N)**. The magenta color represents cells where Ca^2+^ mobilization was induced. **(F–K)** Scale bar: 100 μm; **(L–N)** scale bar: 500 μm. Data are representative of three independent experiments.

### Immunohistochemistry and Confocal Microscopy

Phoenix auditory neuroprogenitors were plated on Matrigel-coated coverslips in 24-well plates. For immunostaining of differentiated cells, isolated neuroprogenitors were cultured 7 days in differentiation media. For immunostaining of auditory progenitors, undissociated neurospheres were left untouched for 4 h in the incubator, in order to attach to the coverslip. Fixation of the samples was performed using a 4% paraformaldehyde solution for 15 min. Specimens were then permeabilized (0.2% Triton-X 100 in PBS 1×) for 30 min at room temperature and incubated overnight at 4°C with the primary antibody diluted in a blocking buffer containing 2% bovine serum albumin and 0.01% Triton-X 100 in PBS. The following day, the cells were rinsed 10 min three times with PBS and incubated for 2 h at room temperature with the secondary antibodies in blocking buffer. The stained samples were washed three times with PBS and mounted on glass slides with Fluoroshield containing DAPI (Sigma–Aldrich). The labeled specimens were visualized using a Super Resolutive confocal laser-scanning microscope (LSM800 Airyscan). During the image acquisition, five *Z*-stack planes were imaged using a 20× or 40× objectives. The final *Z-stack* projection was edited using FIJI (ImageJ-win64, version 1.53a) software.

### RNA Extraction and Real-Time Quantitative Polymerase Chain Reaction

RNA extraction was performed at different stages of differentiation (days 0, 1, 3, and 7) using a Qiagen RNA extraction minikit (Qiagen), according to the manufacturer’s protocol. RNA concentration was determined using a Nanodrop spectrometer, and 500 ng of RNA was used for cDNA synthesis using the Takara PrimeScript RT reagent kit, following manufacturer’s instruction. Real-time PCR was performed using an SYBR green assay on a 7900HT SDS system from ABI. The efficiency of each primer was verified with serial dilutions of cDNA. Relative expression levels were calculated by normalization to the geometric mean of the three housekeeping genes (*Eef1a*, *Tubb*, and *Actb*). The highest normalized relative quantity was arbitrarily designated as a value of 1.0. Fold changes were calculated from the quotient of means of these RNA normalized quantities and reported as ±SEM. The list of primers is displayed in Supplementary Table 2.

### RNA Sequencing

At passages 12, 21, and 36, phoenix auditory neuroprogenitors from three different sources were differentiated in 6-well plates as described above for 7 days. TruSeq ribodepleted stranded mRNA was applied to both progenitors and differentiated cells, in triplicates, to eliminate ribosomic RNA and sequenced using Illumina TruSeq protocol. The sequencing quality control was done with FastQC v.0.11.5. The sequences were mapped with the STAR v.2.7.0 software to the UCSC *Mus musculus* mm10 reference with an average mapping rate of 86.36%. The biological quality control and summarization were performed using the PicardTools v.1.141. The table of counts with the number of reads mapping each gene feature of UCSC mm10 was prepared with HTSeq v0.9p1. The differential expression analysis was performed with the statistical analysis R/Bioconductor package edgeR v.3.26.8. Briefly, the counts were normalized according to the library size and filtered. The genes having a count greater than 1 count per million reads (CPM) in at least four samples were kept for the analysis. The initial number of genes in the set was 24,420, and after the poorly or nonexpressed genes were filtered out, 13,483 genes were left. The differentially expressed gene tests were done with a GLM (general linear model) with a negative binomial distribution, taking into account the pairing of the samples. The *p*-values of differentially expressed gene analysis were corrected for multiple testing error with a 5% FDR (false discovery rate) using the Benjamini–Hochberg procedure. All RNA-sequencing data files were submitted to the ArrayExpress database at EMBL-EBI^1^ under the accession number MTAB-9441. Main differentially expressed gene ontologies were determined using G:profiler^2^. Relevant gene ontologies as identified on G:profiler served as the basis for marker gene classification, and significantly differentially expressed genes (FDR < 0.05) belonging to the following ontologies are shown on the graphs (Figure 4A: cell cycle process GO:0022402; Figure 4B: synapse GO:0045202; Figure 4C: glial cell differentiation GO:0010001; myelination in peripheral nervous system GO:0022011; peripheral nervous system myelin formation GO:0032290, glial cell development GO:0021782, Schwann cell development GO:0014044; and Figure 4D: stem cell differentiation GO:0048863). In addition, the discrimination between types I and II auditory neurons (Figures 4E,F) was done based on recent studies showing auditory neuron subtypes in the spiral ganglia (see Petitpré et al., 2018; Shrestha et al., 2018; Sun et al., 2018). From these references, lists of types I and II specific genes were established and compared to our transcriptome data. Significantly differentially expressed genes (FDR < 0.05) belonging to these lists are shown on the graphs (Figures 4E,F).

### Statistical Analysis

All data were analyzed using one-way analysis of variance followed by Dunnett multiple-comparisons test with GraphPad Prism software (version 8.4.3) unless where stated otherwise in the figure legend. *p* < 0.05 was considered as statistically significant. *p < 0.05, **p < 0.01, ***p < 0.005, ****p < 0.0005.

## Results

### Cochlear Neuroprogenitors From A/J Mice Exhibit High Intrinsic Self-renewal Properties

Sphere-derived auditory neurons provide a convenient cell model for morphological and electrophysiological studies, *in vitro*. However, the propagation potential of auditory neurospheres is generally limited to a few passages before reaching senescence. We isolated cochlear progenitors from two different genetic backgrounds, namely, from C57BL6 and A/J mice P5 postnatal (Figure 1A). As described in a previous report (Oshima et al., 2009), neuroprogenitors from either genetic background formed neurospheres *in vitro*, with no obvious visual differences between strains. However, while C57Bl6 progenitors failed to expand after the third passage (Figures 1C,D), neuroprogenitors from A/J mice (phoenix) exhibited continuous growth up to over 40 passages (Figures 1B,D–F). To determine the relative proportion of proliferating cells, we studied the DNA content of neuroprogenitors, which is indicative of the cell cycle phase (Figures 1D,E; Supplementary Figure 1). Supporting our observations, up to passage 36 and at other passages tested (12 and 21), more than a third of the A/J auditory progenitor pool was engaged in the cell cycle (S or G2M phase) indicating a stable proliferation over passages (Figure 1D, red plot, Figure 1E; Supplementary Figure 1). In contrast, C57Bl6 neuroprogenitors were blocked in G0–G1 phase peak—with a vast majority of quiescent cells (Figure 1D, blue plot). Already following the first passage, we could observe in C57Bl6 cultures empty “hollow” spheres, which became more abundant after the second and third passages (Figure 1C, lower panel). Cells were subjected to several freezing and thawing cycles and sent to Universities of Tübingen (Dr. Müller) and Innsbruck (Dr. Glückert; Figure 2). In three different laboratories, phoenix neuroprogenitors could be easily propagated up to passage 40 (Figure 2B) and, upon growth factor removal, differentiated into auditory neuron–like cells (Figure 2C), expressing BIII-tubulin (Figure 2D). Together, the data demonstrate that phoenix auditory neuroprogenitors have virtually unlimited intrinsic self-renewal properties and can easily endure freeze and thaw cycles. Therefore, cells coming from a single primary source can constitute a nearly unlimited source of auditory neurons.

### Transcriptomic Profile of Phoenix Auditory Spiral Ganglion Differentiated Cells

In order to characterize molecular events underlying differentiation of phoenix neuroprogenitors, we performed a transcriptomic comparison of neuroprogenitor spheres and neurons differentiated for 7 days (D7; Figures 3, 4). Cells were taken at different passage numbers (paired-samples progenitors vs. differentiated at P12, P21, and P36) in order to detect passage-dependent specific changes (Figure 3). Upon growth factor removal and phoenix differentiation, 3,180 genes were differentially expressed (FDR < 0.05); among them 1,663 were upregulated and 1,517 were downregulated in differentiated neurons when compared to the progenitors (Figure 3A). Accordingly, the multidimensional scaling plot showed a clear segregation between neuroprogenitors and differentiated cells (Figure 3B). Although collected at different numbers of passages (from P12 to P36), triplicate samples from neuroprogenitors and from differentiated cells were well clustered, suggesting a conserved pattern of gene expression with passages. Similarly, the heatmap representation of differentially expressed genes revealed comparable patterns of gene expression between triplicates (Figure 3C). Upon differentiation, canonical neuronal markers were upregulated [about 10-fold induction of BIII-tubulin (FDR = 0.038), 30-fold induction of parvalbumin (FDR = 0.029)] together with peripheral nervous system marker genes such as peripherin (10-fold induction; FDR = 0.009) or gamma synuclein (181-fold induction; FDR = 0.002; Figure 4B). Interestingly, both type I–related (Figure 4F) and type II–related (Figure 4E) auditory neuron markers were induced. Strong induction of glia-related genes was also observed, demonstrating the ability of phoenix neuroprogenitors to differentiate in both neuron and glial cells (Figure 4C). Significantly induced genes were related to peripheral nervous system myelin formation (*Cntnap1*, *Fa*2*h*, *Itgb4*, *Lgi4*, *Ntrk2*, *Sirt2*), glial cell development (*Pdgfb*, *Miat*, *Mmp24*, *Lgi4*, *Gap43*), or Schwann cell development (*Lgi4*). In contrast, genes related to progenitor cells and to the cell cycle were dramatically downregulated (Figures 4A,D). Accordingly, main downregulated gene ontologies referred to the cell cycle (*p* = 10^−63^), whereas upregulated ontologies were related to neural cell body (*p* = 10^−7^), synapse (*p* = 10^−8^), axon (*p* = 10^−11^) and neuron projections (*p* = 10^−16^; Figure 4G). In a further attempt to characterize phoenix cells, we compared our transcriptomic dataset to a recently published dataset on mouse auditory neurons comparing different ages (GSE132925), from (Li et al., 2020; Supplementary Figure 2). We assumed that differentially expressed genes upon SGN postnatal development should, to an extent, overlap with differentially expressed genes during phoenix differentiation. We therefore compared differentially expressed genes between primary auditory neurons extracted from P1 postnatal mouse vs. P30 mouse and upon phoenix differentiation (neurospheres vs. D7 differentiated cells). The results highlighted 66 overlapping genes between phoenix and primary auditory neurons that were significantly upregulated, respectively, upon differentiation or postnatal development (FDR < 0.05; Supplementary Figure 2A). Conversely, 128 genes were significantly enriched in progenitors and P1 postnatal SGN (Supplementary Figure 2B). By using hypergeometric probability distribution, we observed significant overlap of differentially expressed genes between the two comparisons (*p* = 0.03420227). This *p*-value indicates a similar trend in the pattern of gene regulation in phoenix upon differentiation and in primary auditory neurons during postnatal development. For instance, dynamically expressed genes include auditory neuron marker GATA3, which expression was previously shown to peak in developing neurons and decrease in mature neurons (Nishimura et al., 2017). Together, the data demonstrate that at any passage, phoenix auditory neuroprogenitors are able to differentiate and express markers from the main mature cell types of the spiral ganglion region, namely, types I and II auditory neurons and glial cells. Furthermore, phoenix cells can recapitulate, to some extent, postnatal auditory neuron development and maturation as observed *in vivo* (Nishimura et al., 2017; Li et al., 2020).

### Sphere-Derived Phoenix Auditory Spiral Ganglion Differentiated Cells Express Markers of Auditory Neurons

Phoenix auditory progenitors exhibit virtually unrestricted propagation potential. To assess whether this unique feature affects their phenotype, we addressed several progenitors and mature neuron markers by quantitative polymerase chain reaction (qPCR) and immunostaining on phoenix neuroprogenitor spheres (D0) differentiating (D1 and D3) and differentiated neurons (D7; Figure 5). Phoenix auditory neuroprogenitors expressed classical markers of neural stem cells Sox2 and nestin (Figures 5A,B; Supplementary Table 2). Furthermore, consistent with previous observations (Figure 1), the proliferation marker Ki67 was strongly expressed in neuroprogenitor cells (Figure 5C). Interestingly, the expression of stemness/proliferation markers was no longer detectable in D7 differentiated auditory neurons (Figures 5D–F). Conversely, while the expression of BIII-tubulin (TuJ1) was below detection levels in progenitor spheres (Figures 5A–C), high expression was observed in differentiated cells (Figures 5D–F, green). In addition, in differentiated cells, we could identify glial cells expressing glial fibrillary acidic protein (GFAP; Figure 5J) and cells expressing markers of mature neurons, including Map2 (Figure 5K) and the sensory neuron marker Islet1 (Figure 5L). Note that these markers were absent in neurospheres (Figures 5G–I). Studying the time course of differentiation, we observed a time-dependent increase in mRNA encoding for BIII-tubulin (Figure 5M), peripherin (Figure 5N), TrkB (Figure 5O), and Islet1 (Figure 5P). Gene induction was already significant after 24 h of differentiation; however, highest expression was reached after 7 days of differentiation. Interestingly, this gradual increase in neuronal marker expression upon differentiation was correlated with similar increase in the glutamatergic response (Figures 5Q,R). In this respect, we studied Ca^2+^ transients mediated by glutamate (0.1–100 μM). At both 3 and 7 days of differentiation, we revealed glutamate-evoked increase in Ca^2+^ mobilization, which was comparable in amplitude and EC_50_ (within the micromolar range; Figures 5Q,R). Remarkably, in D7 differentiated cells, both amplitude and EC_50_ of glutamate-induced Ca^2+^ responses remained unchanged with the passage number (Figure 5S). However, in auditory neuroprogenitors (D0) and early differentiating cells (D1), the glutamatergic function was markedly decreased, indicating that the phenotypic switch between self-renewal and differentiation of the phoenix auditory neuroprogenitors is easily inducible at any passage. Differentiated phoenix auditory neurons exhibit markers and functional features of mature auditory neurons. Moreover, the differentiated phoenix spheres show a stable phenotype over all passages (up to 40) investigated.

### Induction of Glial Marker Expression Upon Differentiation

The transcriptomic analysis demonstrated significant induction of both peripheral and central nervous system glia markers (Figure 4E). Glial induction was confirmed at the protein level (Figure 6) where several cells appeared positive for the neuronal intermediate filament marker S-100 upon differentiation (Figures 6A–D; Supplementary Figure 3). Note that some cells express both characteristics (arrows) that classify them in a differentiation state between glial cell or a neuron. We used midmodiolar cross-section of mouse pups from the same age as reference, showing wide expression of S100 protein in the spiral ganglion (Figure 6D). Fibrocytes in the spiral ligament, glia cells around the SGN, and glia cells in the central cochlear nerve are also expressing this calcium-binding protein. The data also show the expression of glutamine synthetase, which is usually restricted to the small satellite glia cells that surround bigger SGNs (Figure 6E). Expression of glutamine synthetase was confirmed in satellite glial cells of P5 mouse pups spiral ganglion sections (Figure 6F). GAP-43 immunoreactivity peaks along growing neurites (Figure 6G) and is expressed in both neuronal and glial cells. Altogether, the data show significant glia cells induction, with both satellite and Schwann cell markers being expressed in differentiated phoenix cells.

### Firing Properties of Phoenix-Derived Auditory Neuronal Cells

To further explore the excitatory function of differentiated phoenix SGCs, we studied Ca^2+^ mobilization in response to several physiological stimuli, namely glutamate, ATP, and kainate (Figure 7). Robust Ca^2+^ transients were induced in D7 differentiated SGCs upon glutamate (Figure 7A) and ATP (Figure 7B) stimulation with respective EC_50_ values of 1.16 and 0.43 μM (Figures 7D,E). However, kainate failed to induce Ca^2+^ excitatory transmission in phoenix SGCs (Figures 7C,D). Ionomycin, an ionophore facilitating the transport of divalent cations across cell membranes, and thapsigargin, inhibiting the SERCA pump and therefore the transfer of Ca^2+^ toward endoplasmic reticulum, were used as positive controls (Figure 7D, Supplementary Figure 4). The excitatory pattern of phoenix SGCs (Figures 7F–H) was compared with human fetal sphere-derived ones (Figures 7I–K) and mouse spiral ganglion explants (Figures 7L–N) using live Ca^2+^ imaging (Supplementary Videos). Similarly to phoenix SGCs, human fetal sphere-derived SGCs and mouse spiral ganglion explants exhibited glutamatergic and purinergic evoked Ca^2+^ transients. However, the cultures did not respond to kainate (Figures 7H,K,N). Together, our data demonstrate similar excitatory properties of both human and phoenix auditory SGC. Therefore, at any passage and following differentiation, phoenix auditory SGCs can resemble the auditory neuron physiology.

## Discussion

We hereby provide a first observation and an in-depth analysis of auditory neuroprogenitors derived from the A/J mouse spiral ganglion with unparalleled self-renewal capacities beyond 40 passages. In addition to the self-renewal, the so-called phoenix neurospheres can be differentiated at any passage and after multiple freezing–thawing cycles into neuronal and glial cells with robust expression of specific markers and functional maturity of glutamatergic and purinergic pathways, similar to those observed from fetal human auditory neurons and mouse spiral ganglion organotypic explants. To confirm this unexpected finding, crucial propagation and differentiation experiments were successfully repeated in the inner ear laboratories of the University of Innsbruck (Austria) and Tübingen (Germany).

From a 3R perspective, the phoenix sphere-derived auditory neurons constitute a good alternative to circumvent the very limited number of auditory neurons available in the cochlea. By using phoenix neurospheres, the number of differentiated auditory neurons per one animal can be amplified beyond 1 trillion-fold (theoretically >10^12^-fold at passage 40). Furthermore, phoenix spheres can be easily frozen and thawed and therefore used as regular cell lines substantially cutting down on numbers of animals needed for experimentation. To the best of our knowledge, existing protocols have reported the possibility to propagate sphere-derived auditory neuroprogenitors only for a few passages, with already significant evidence of senescence and stemness loss in tertiary spheres (Senn et al., 2007; Diensthuber et al., 2014). These auditory neuroprogenitor spheres reach the limits of their intrinsic ability to propagate and become senescent very rapidly. This intrinsic propagation barrier is a major limitation of such an *in vitro* model. We generated sphere-derived auditory neuroprogenitors with virtually no self-renewal restrictions.

Transcriptomic data showed a dramatic change in gene expression upon induction of differentiation (Figures 3, 4). Growth factor removal led to a switch from a proliferative to a maturation state with strong decrease in cell cycle and progenitor-related genes and upregulation of mature neuron markers (Oshima et al., 2007). Interestingly, both types I and II neural markers, together with glial cell markers, were induced in the differentiated population, demonstrating that phoenix neuroprogenitors are able to reproduce the cellular diversity present in the spiral ganglia (Petitpré et al., 2018; Shrestha et al., 2018; Sun et al., 2018). Note that even after many passages, a significant proportion of the phoenix progenitors differentiated toward glial cells, expressing GFAP (Figures 4C, 5J), including both Schwann (S100, PLP1) and satellite glial cells (glutamine synthetase; Figure 6), a feature previously observed for SGN progenitors from other mouse strains (Oshima et al., 2007; Martinez-Monedero et al., 2008; Diensthuber et al., 2014; McLean et al., 2016; Moon et al., 2018). Whether the glial cells and neurons are originating from a distinct progenitor subtype in the phoenix neurosphere culture remains to be investigated. However, data from the literature have demonstrated the existence of a common neural and glial progenitor (PLP1^+^) in the spiral ganglia (McLean et al., 2016). Similarly to phoenix neuroprogenitor, PLP1^+^ cells were shown to give neurons and glial cells, also expressing markers of glial central nervous system. Identifying and comparing the phoenix common progenitor to the PLP1^+^ spiral ganglion progenitors could provide important insight into spiral ganglion cell development and renewal.

At the functional level, however, most of the newly formed neurons did not respond to kainate. This indicates type I auditory neuron firing properties (Petitpré et al., 2018) as kainate receptors have been shown to be mainly located at the OHC/type II auditory neuron synapse, possibly mediating efferent signal to outer hair cells (OHC; Fujikawa et al., 2014). The absence of response to kainate does not appear to be a specific feature of phoenix auditory neurons. We observed a similar firing pattern in human sphere-derived auditory neurons and mouse spiral ganglion explants (Figures 7I–N; Supplementary Videos). The absence of response to kainate might be explained by the relatively early maturation stage of the tested neurons: fetal sphere-derived human auditory neurons and SGN explants of P5 mouse pups. In contrast, all differentiated cells exhibited robust glutamatergic and purinergic responses (Figure 7). These data are consistent with the glutamatergic nature of the auditory synapse, whereas the purinergic signal was demonstrated to be involved in the process of auditory neuron maturation in young animals (Jovanovic et al., 2017).

The A/J mouse is a commonly used strain for studies on lung carcinogenesis since it has been reported to develop tumors with high prevalence upon carcinogen exposure (Witschi, 2005). Whether this particularity is of importance in the current context remains to be investigated; however, the cell cycle of phoenix cells appeared to be normal with no additional DNA peaks, indicating a possible aneuploidy or genomic instability, arguing for relative genomic stability in these cells even at a high passage number (Supplementary Figure 1). In line with this observation, differentiated phoenix cells exhibited remarkably stable phenotype over passages (Figures 3, 4), although smaller chromosomic rearrangements have not been experimentally excluded and remain possible at a high passage number (Lefort et al., 2008) or following freezing and thawing cycles. Other *in vitro* models of auditory neurons have been previously described (Kwan et al., 2015; Walters et al., 2015). However, in these models, an extrinsic factor such as genetic transformation (Kwan et al., 2015) or conditional reprogramming (Walters et al., 2015) of the progenitors is required to bypass the natural self-renewal barrier of these cells. Genomic instability is a relatively frequent phenomenon following cell immortalization (Bikkul et al., 2019), which is no longer needed if the A/J mouse is used. Yet, it is difficult to understand why auditory neuroprogenitors from the A/J mouse strain exhibit such high self-renewal ability compared to previously described models. In mice, proliferation of SGN progenitors is typically observed during the first postnatal week, but rapidly decreases with age to virtually no regeneration in adults (Oshima et al., 2007; Moon et al., 2018; Senn et al., 2020). The failure of the adult inner ear to regenerate following injury might be explained by the low intrinsic self-renewal ability of mammalian auditory neuroprogenitors. Paradoxically, the A/J mouse line, from which arise phoenix neuroprogenitors, has recently been described to exhibit early onset of hearing loss with strong auditory neuropathy (Rousset et al., 2020). Therefore, neuroprogenitor self-renewal followed by regeneration of auditory neurons is unlikely in this model, as adult animals rapidly develop hearing loss with complete deafness at the age of 3 months. At least any regenerative attempt would not be functionally effective (Rousset et al., 2020). Why auditory neuroprogenitors from A/J mice are able to propagate nearly indefinitely *in vitro* but not *in vivo* remains to be elucidated. Hair cell progenitors in mice and humans have similar growth properties compared to auditory neurospheres *in vitro*, exhibiting very limited propagation potential (Senn et al., 2020). Remarkably, self-renewal capacities as observed in spiral ganglion cells from A/J mice did not seem to be conserved by hair cell progenitors (not shown). Conditional reprograming toward pseudoimmortalization of murine hair cell progenitors was shown to enhance telomerase expression and progenitors propagation for up to 10 passages (Walters et al., 2015). As a possible mechanism, an epigenetic-switch related to the *in vitro* culture conditions as previously observed for hair cell progenitors could potentiate the intrinsic self-renewal capacities of the neurospheres and possibly explain this paradox. However, our standardized culture conditions were equivalent to those described in previous reports (Oshima et al., 2009). The evidence points clearly toward an intrinsic property of phoenix spheres, as in strictly comparable culture conditions, we were not able to meaningfully propagate auditory neuroprogenitors from C57Bl6/J (Figure 1C) and from human fetal auditory neurons (not shown) beyond three to four passages. Further understanding of this intrinsic feature of phoenix auditory neuroprogenitors may have important consequences in regenerative medicine and requires additional investigation.

In conclusion, to the best of our knowledge, we describe the first mammalian auditory neuroprogenitor model with nearly unrestricted self-renewal capacities. Phoenix auditory progenitors constitute a convenient and robust model for the study of many aspects of the cochlear pathophysiology and its development, modeling the cellular complexity of spiral ganglia *in vitro*. Large quantities of auditory neuroprogenitors can be produced from a single A/J mouse; frozen, stored, and thawed upon demand; and differentiated at any time needed into functional auditory neuronal cells for use in a variety of experiments including high-throughput screening of regenerative and otoprotective compounds or studies aiming at improving the auditory neuron: cochlear implant–electrode interface through induced and directed neuronal growth (Senn et al., 2017). By substantially reducing the numbers of animals needed to obtain significant quantities of auditory neurons *in vitro*, phoenix auditory neuroprogenitors improve cost-effectiveness for research in the field and are fully in line with the 3R principles.

## Data Availability Statement

The datasets presented in this study can be found in online repositories. The names of the repository/repositories and accession number(s) can be found below: https://www.ebi.ac.uk/arrayexpress/experiments/E-MTAB-9441/.

## Ethics Statement

The studies involving human participants were reviewed and approved by Commission Cantonale d’Ethique de la Recherche sur l’être humain (CCER). The patients/participants provided their written informed consent to participate in this study. The animal study was reviewed and approved by Veterinary office and the Commission for Animal experimentation of the Canton of Geneva, Switzerland, authorization number GE/189/17.

## Author Contributions

FR and PS: conceptualization. FR, GN-S and SI: data curation. FR, VK, RS, DS, GN-S, SI and RG: data analysis. FR, VK, RS, DS, MC, SF, AE and AM: investigation. K-HK and MM: resources. SF, DS, FR, RG and MM: validation. FR and RS: visualization. PS, FR, HL, RG and MM: funding acquisition. FR, VK, RG and PS: writing. SF, DS and FV: review and editing. All authors contributed to the article and approved the submitted version.

## Conflict of Interest

The authors declare that the research was conducted in the absence of any commercial or financial relationships that could be construed as a potential conflict of interest.

## Footnotes

^1^www.ebi.ac.uk/arrayexpress

^2^https://biit.cs.ut.ee/gprofiler/gost

## References

[B1] Bikkul M. U. Faragher G. A. R. Worthington G. Meinke P. Kerr R. W. A. Sammy A.. (2019). Telomere elongation through hTERT immortalization leads to chromosome repositioning in control cells and genomic instability in Hutchinson-Gilford progeria syndrome fibroblasts, expressing a novel SUN1 isoform. Genes Chromosomes Cancer 58, 341–356. 10.1002/gcc.22711 30474255 PMC6590296

[B2] Bixenstine P. J. Maniglia M. P. Vasanji A. Alagramam K. N. Megerian C. A. (2008). Spiral ganglion degeneration patterns in endolymphatic hydrops. Laryngoscope 118, 1217–1223. 10.1097/MLG.0b013e31816ba9cd 18364591

[B3] Chen W. Jongkamonwiwat N. Abbas L. Eshtan S. J. Johnson S. L. Kuhn S.. (2012). Restoration of auditory evoked responses by human ES-cell-derived otic progenitors. Nature 490, 278–282. 10.1038/nature11415 22972191 PMC3480718

[B4] Corwin J. T. Cotanche D. A. (1988). Regeneration of sensory hair cells after acoustic trauma. Science 240, 1772–1774. 10.1126/science.3381100 3381100

[B6] Diensthuber M. Zecha V. Wagenblast J. Arnhold S. Edge A. S. Stover T. (2014). Spiral ganglion stem cells can be propagated and differentiated into neurons and glia. Biores Open Access 3, 88–97. 10.1089/biores.2014.0016 24940560 PMC4048968

[B7] Fujikawa T. Petralia R. S. Fitzgerald T. S. Wang Y.-X. Millis B. Morgado-Diaz J. A.. (2014). Localization of kainate receptors in inner and outer hair cell synapses. Hear. Res. 314, 20–32. 10.1016/j.heares.2014.05.001 24858010 PMC4107312

[B8] Jovanovic S. Radulovic T. Coddou C. Dietz B. Nerlich J. Stojilkovic S. S.. (2017). Tonotopic action potential tuning of maturing auditory neurons through endogenous ATP. J. Physiol. 595, 1315–1337. 10.1113/JP273272 28030754 PMC5309364

[B9] Kujawa S. G. Liberman M. C. (2009). Adding insult to injury: cochlear nerve degeneration after “temporary” noise-induced hearing loss. J. Neurosci. 29, 14077–14085. 10.1523/JNEUROSCI.2845-09.2009 19906956 PMC2812055

[B10] Kwan K. Y. Shen J. Corey D. P. (2015). C-MYC transcriptionally amplifies SOX2 target genes to regulate self-renewal in multipotent otic progenitor cells. Stem Cell Reports 4, 47–60. 10.1016/j.stemcr.2014.11.001 25497456 PMC4297878

[B11] Lefort N. Feyeux M. Bas C. Feraud O. Bennaceur-Griscelli A. Tachdjian G.. (2008). Human embryonic stem cells reveal recurrent genomic instability at 20q11.21. Nat. Biotechnol. 26, 1364–1366. 10.1038/nbt.1509 19029913

[B14] Li X. Aleardi A. Wang J. Zhou Y. Andrade R. Hu Z. (2016). Differentiation of spiral ganglion-derived neural stem cells into functional synaptogenetic neurons. Stem Cells Dev. 25, 803–813. 10.1089/scd.2015.0345 27021700 PMC4870608

[B12] Li C. Li X. Bi Z. Sugino K. Wang G. Zhu T.. (2020). Comprehensive transcriptome analysis of cochlear spiral ganglion neurons at multiple ages. eLife 9:e50491. 10.7554/eLife.50491 31913118 PMC7299348

[B15] Martinez-Monedero R. Yi E. Oshima K. Glowatzki E. Edge A. S. B. (2008). Differentiation of inner ear stem cells to functional sensory neurons. Dev. Neurobiol. 68, 669–684. 10.1002/dneu.20616 18278797

[B16] McLean W. J. McLean D. T. Eatock R. A. Edge A. S. (2016). Distinct capacity for differentiation to inner ear cell types by progenitor cells of the cochlea and vestibular organs. Development 143, 4381–4393. 10.1242/dev.139840 27789624 PMC5201044

[B17] Michelson R. P. Merzenich M. M. Pettit C. R. Schindler R. A. (1973). A cochlear prosthesis: further clinical observations; preliminary results of physiological studies. Laryngoscope 83, 1116–1122. 10.1288/00005537-197307000-00015 4719358

[B18] Moon B.-S. Ammothumkandy A. Zhang N. Peng L. Ibrayeva A. Bay M.. (2018). The presence of neural stem cells and changes in stem cell-like activity with age in mouse spiral ganglion cells *in vivo* and *in vitro*. Clin. Exp. Otorhinolaryngol. 11, 224–232. 10.21053/ceo.2018.00878 30309200 PMC6222184

[B19] Moser T. Starr A. (2016). Auditory neuropathy—neural and synaptic mechanisms. Nat. Rev. Neurol. 12, 135–149. 10.1038/nrneurol.2016.10 26891769

[B20] Nishimura K. Noda T. Dabdoub A. (2017). Dynamic expression of Sox2, Gata3, and Prox1 during primary auditory neuron development in the mammalian cochlea. PLoS One 12:e0170568. 10.1371/journal.pone.0170568 28118374 PMC5261741

[B21] Olusanya B. O. Davis A. C. Hoffman H. J. (2019). Hearing loss: rising prevalence and impact. Bull. World Health Organ. 97, 646A–646A. 10.2471/BLT.19.224683 31656325 PMC6796666

[B22] Oshima K. Grimm C. M. Corrales C. E. Senn P. Martinez Monedero R. Geleoc G. S.. (2007). Differential distribution of stem cells in the auditory and vestibular organs of the inner ear. J. Assoc. Res. Otolaryngol. 8, 18–31. 10.1007/s10162-006-0058-3 17171473 PMC2538418

[B23] Oshima K. Senn P. Heller S. (2009). Isolation of sphere-forming stem cells from the mouse inner ear. Methods Mol. Biol. 493, 141–162. 10.1007/978-1-59745-523-7_9 18839346 PMC2861714

[B24] Petitpré C. Wu H. Sharma A. Tokarska A. Fontanet P. Wang Y.. (2018). Neuronal heterogeneity and stereotyped connectivity in the auditory afferent system. Nat. Commun. 9:3691. 10.1038/s41467-018-06033-3 30209249 PMC6135759

[B25] Roccio M. Perny M. Ealy M. Widmer H. R. Heller S. Senn P. (2018). Molecular characterization and prospective isolation of human fetal cochlear hair cell progenitors. Nat. Commun. 9:4027. 10.1038/s41467-018-06334-7 30279445 PMC6168603

[B26] Rousset F. Nacher-Soler G. Coelho M. Ilmjarv S. Kokje V. B. C. Marteyn A.. (2020). Redox activation of excitatory pathways in auditory neurons as mechanism of age-related hearing loss. Redox Biol. 30:101434. 10.1016/j.redox.2020.101434 32000019 PMC7016250

[B27] Ryals B. M. Rubel E. W. (1988). Hair cell regeneration after acoustic trauma in adult Coturnix quail. Science 240, 1774–1776. 10.1126/science.3381101 3381101

[B28] Senn P. Mina A. Volkenstein S. Kranebitter V. Oshima K. Heller S. (2020). Progenitor cells from the adult human inner ear. Anat. Rec. 303, 461–470. 10.1002/ar.24228 31489779 PMC7064943

[B29] Senn P. Oshima K. Teo D. Grimm C. Heller S. (2007). Robust postmortem survival of murine vestibular and cochlear stem cells. J. Assoc. Res. Otolaryngol. 8, 194–204. 10.1007/s10162-007-0079-6 17334849 PMC2538352

[B30] Senn P. Roccio M. Hahnewald S. Frick C. Kwiatkowska M. Ishikawa M.. (2017). NANOCI-nanotechnology based cochlear implant with gapless interface to auditory neurons. Otol. Neurotol. 38, e224–e231. 10.1097/MAO.0000000000001439 28806330 PMC5559190

[B31] Shrestha B. R. Chia C. Wu L. Kujawa S. G. Liberman M. C. Goodrich L. V. (2018). Sensory neuron diversity in the inner ear is shaped by activity. Cell 174, 1229.e17–1246.e17. 10.1016/j.cell.2018.07.007 30078709 PMC6150604

[B32] Sun S. Babola T. Pregernig G. So K. S. Nguyen M. Su S. M.. (2018). Hair cell mechanotransduction regulates spontaneous activity and spiral ganglion subtype specification in the auditory system. Cell 174, 1247.e15–1263.e15. 10.1016/j.cell.2018.07.008 30078710 PMC6429032

[B33] Vartiainen E. Karjalainen S. Kärjä J. (1985). Auditory disorders following head injury in children. Acta Otolaryngol. 99, 529–536. 10.3109/00016488509182257 4024901

[B34] Walters B. J. Diao S. Zheng F. Walters B. J. Layman W. S. Zuo J. (2015). Pseudo-immortalization of postnatal cochlear progenitor cells yields a scalable cell line capable of transcriptionally regulating mature hair cell genes. Sci. Rep. 5:17792. 10.1038/srep17792 26639154 PMC4671002

[B35] Warchol M. E. Lambert P. R. Goldstein B. J. Forge A. Corwin J. T. (1993). Regenerative proliferation in inner ear sensory epithelia from adult guinea pigs and humans. Science 259, 1619–1622. 10.1126/science.8456285 8456285

[B36] Witschi H. (2005). The complexities of an apparently simple lung tumor model: the A/J mouse. Exp. Toxicol. Pathol. 57, 171–181. 10.1016/j.etp.2005.05.005 16092725

[B37] Xiong B. Liu Z. Liu Q. Peng Y. Wu H. Lin Y.. (2019). Missed hearing loss in tinnitus patients with normal audiograms. Hear. Res. 384:107826. 10.1016/j.heares.2019.107826 31683074

