# Supplementary material for: Intrinsically Self-renewing Neuroprogenitors From the A/J Mouse Spiral Ganglion as Virtually Unlimited Source of Mature Auditory Neurons
Source: Front Cell Neurosci. 2020 Dec 9;14:395. doi: 10.3389/fncel.2020.599152 (PMC7761749; doi:10.3389/fncel.2020.599152)
Supplement: Supplementary file 1 [file Presentation_1.pdf]

Supplementary Figure 1

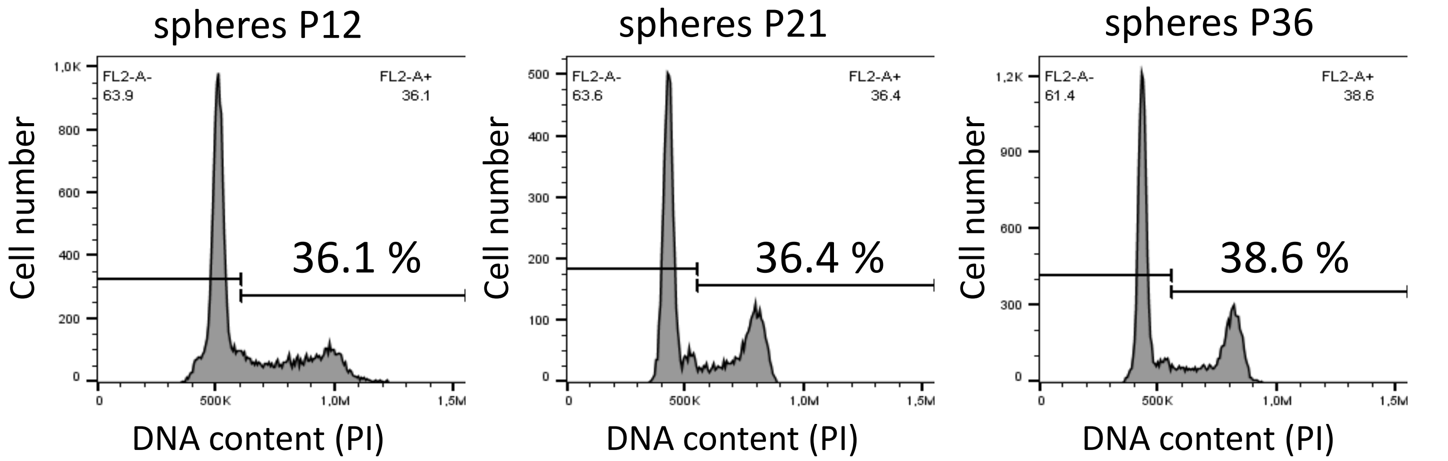

Supplementary Figure 1. phoenix auditory neuroprogenitors exhibit stable proportion of proliferating cells over passages. DNA content was quantified by flow cytometry in phoenix auditory neuroprogenitors using propidium iodide staining respectively at passage A) 12, B) 21 and C) 36. At any passage tested, percentage of proliferating cells (S-G2M cell cycle phase) was between 35 and 40.

Supplementary Figure 2

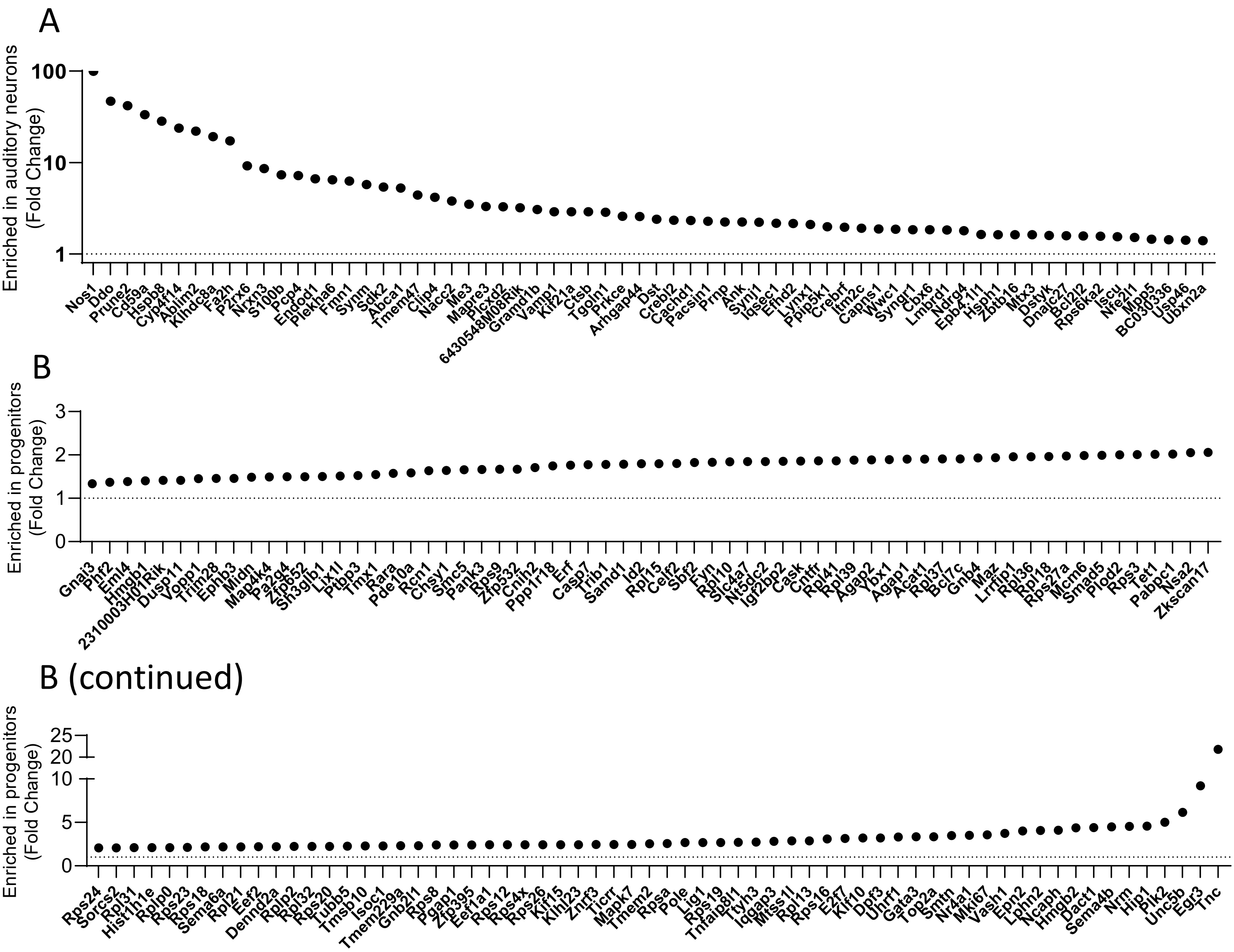

Supplementary Figure 2. Overlapping genes differentially expressed during primary auditory neuron postnatal development (*in vivo*) and phoenix differentiation. A) Comparison of differentially expressed genes in the publicly available dataset from Li et al. 2020 (GSE132925) and phoenix transcirptome dataset. Assuming that differentially expressed genes upon SGN development should significantly overlap with differentially expressed genes upon phoenix differentiation, we looked for differentially expressed genes between primary auditory neurons extracted from P1 postnatal mouse vs. P30 mouse. A) 66 genes shown to be upregulated in Li et al. between P1 and P30 auditory neuron that were also upregulated in phoenix differentiated neurons compared to neuroprogenitors (FDR < 0.05). The graph shows the relative enrichment of these genes in phoenix auditory neurons vs. phoenix neuroprogenitors. B) 128 overlapping genes between phoenix and primary auditory neurons that were significantly downregulated respectively upon differentiation or post-natal development (FDR < 0.05). Graph shows the enrichment of these genes in phoenix auditory neuroprogenitors.

Supplementary Figure 3

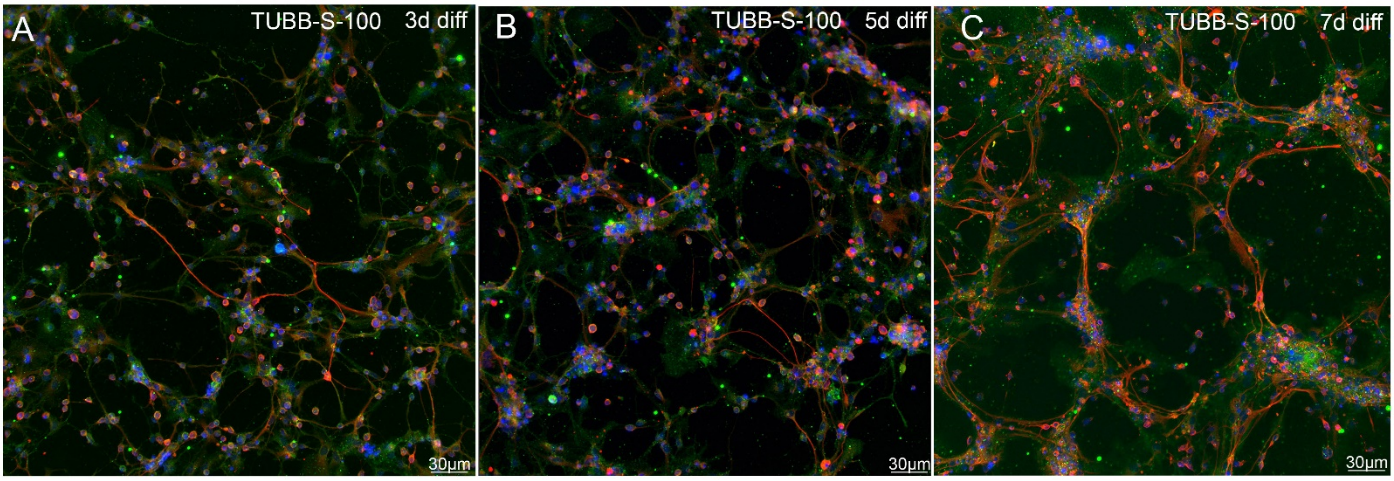

Supplementary Figure 2. Phoenix differentiation towards glial cells and neurons. A-C) Beta-III-tubulin (red) /S-100 (green) staining at different time points of differentiation. 3 days in differentiation medium (A) results in only few neurons that show neurite outgrowth, most cells are S-100 positive non-neural cells. The relative proportion of neuron-like cells increases after 5 days (B) and more neurites become visible. 7 days in differentiation medium (C) leads to cell clustering with long neurites. S-100 cells diminish relatively but still embrace neurite pathways.

Supplementary Figure 4

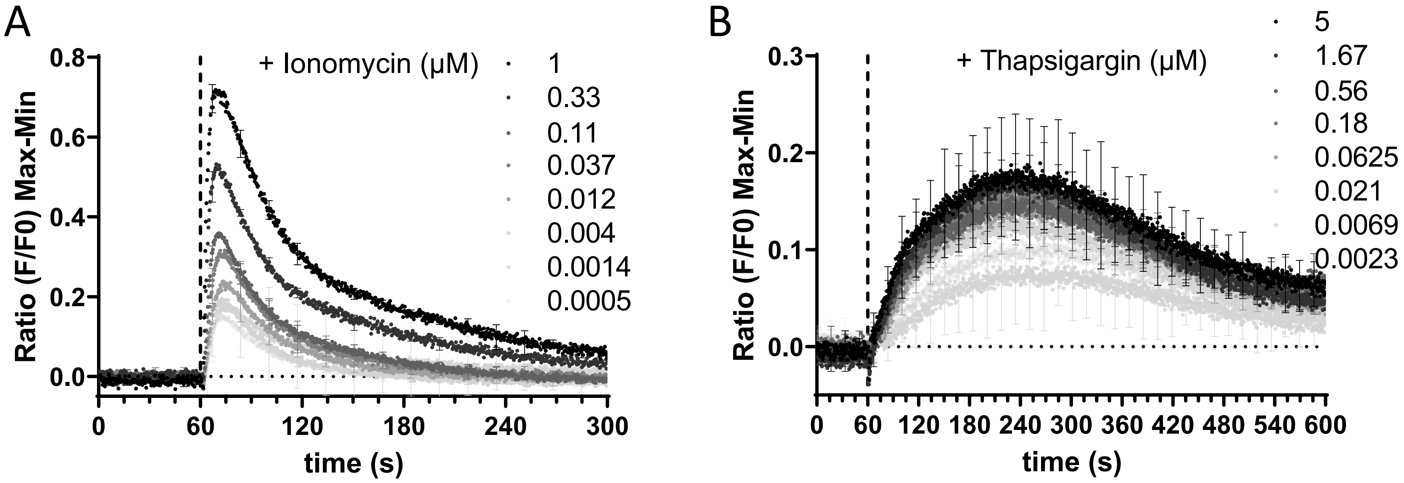

Supplementary Figure 3. Effect of Ionomycin and Thapsigagin on Ca^{2+} transients in Phoenix auditory neurons. A-C) Kinetics of Ca2+ transients induced by A) Ionomycin and B) Thapsigargin in phoenix auditory neurons after 7 days of differentiation. Phoenix auditory neurons were loaded with Ca^{2+} sensitive ratiometric probe (FLUO-8) and stimulated with increasing concentration of Ionomycin (0.5nM to 1µM) and Thapsigagin (0.0023-5µM). Data represent the average +/- SEM of 3 independent experiments.

Supplementary Table 1

Name	Primary/Secondary	Specie, mono/poly-clonal	Supplier	Reference	Dilution
**Alexa fluor 488**	Secondary	Polyclonal Goat IgG	Abcam	ab150129	1:1000
**Alexa fluor 488**	Secondary	Polyclonal Rabbit IgG	ThermoFisher	A-21206	1:1000
**Alexa fluor 555**	Secondary	Polyclonal Mouse IgG	ThermoFisher	A-31570	1:1000
**β III-Tubulin**	Primary	Monoclonal mouse IgG2b	Sigma	T8660	1:1000
**β III-Tubulin**	Primary	Polyclonal Rabbit IgG	BioLegend	PRB435P	1:2000
**β III-Tubulin**	Primary	Monoclonal Rabbit	Abcam	ab52623	-
**GFAP**	Primary	Monoclonal mouse IgG1	Millipore	MAB360	1:2000
**S-100**	Primary	mouse monoclonal IgG_{2a}	Santa Cruz	sc-393919	-
**Islet1**	Primary	Monoclonal αRabbit	Abcam	ab109517	1:250
**Ki67**	Primary	Monoclonal αmouse IgG1	Chemicon	MAB4190	1:100
**Map2**	Primary	Monoclonal αmouse IgG1	Sigma Alrich	M4403	1:1000
**Nestin**	Primary	monoclonal αMouse IgG1	Chemicon	MAB353	1:1000
**Sox2**	Primary	Monoclonal αMouse 20G5	Abcam	ab171380	1:200
**GAP43**	Primary	Polyclonal Rabbit IgG	Novus Biologicals	NB300-143	-

Supplementary table 1. List of primary and secondary antibodies used in the study

Supplementary Table 2

Markers used in Fig. 5 and 6	expressing cells in spiral ganglia	Developmental stage	Example of references
**Sox2**	AN/neuroblasts	Embryo / early postnatal	[1]
	Glial cells	Adult	
**Nestin**	Neuronal progenitors	Embryo+++/ Adult+	[2]
**Ki67**	Proliferating cells	Embryo+++/ Adult+	[3]
**Tuj1**	AN	Early postnatal / adult	[4]
**Map2**	AN	Early postnatal / adult	[5]
**Islet1**	AN?	Embryo / early postnatal	[6]
**GFAP**	PNS Glia upon injury	Adult	[7]
	or in vitro	First week postnatal	[8,9,10]
**S100**	Satellite and Schwann cells	Early postnatal / adult	Fig. 6D; [11],[12]
**GS**	Satellite glial cells	Fetal<br>Early postnatal / adult	[13]<br>Fig. 6F; [14]
**Gap43**	AN, Glia	Embryo+++/ Adult+	[15]

1 Nishimura, K., Noda, T. & Dabdoub, A. Dynamic Expression of Sox2, Gata3, and Prox1 during Primary Auditory Neuron Development in the Mammalian Cochlea. *PLoS One* **12**, e0170568, doi:10.1371/journal.pone.0170568 (2017).

2 Chow, C. L. *et al.* Evaluation of Nestin Expression in the Developing and Adult Mouse Inner Ear. *Stem Cells Dev* **25**, 1419-1432, doi:10.1089/scd.2016.0176 (2016).

3 Moon, B. S. *et al.* The Presence of Neural Stem Cells and Changes in Stem Cell-Like Activity With Age in Mouse Spiral Ganglion Cells In Vivo and In Vitro. *Clin Exp Otorhinolaryngol* **11**, 224-232, doi:10.21053/ceo.2018.00878 (2018).

4 Perny, M. *et al.* The Severity of Infection Determines the Localization of Damage and Extent of Sensorineural Hearing Loss in Experimental Pneumococcal Meningitis. *J Neurosci* **36**, 7740-7749, doi:10.1523/JNEUROSCI.0554-16.2016 (2016).

5 Puligilla, C., Dabdoub, A., Brenowitz, S. D. & Kelley, M. W. Sox2 induces neuronal formation in the developing mammalian cochlea. *J Neurosci* **30**, 714-722, doi:10.1523/JNEUROSCI.3852-09.2010 (2010).

6 Radde-Gallwitz, K. *et al.* Expression of Islet1 marks the sensory and neuronal lineages in the mammalian inner ear. *J Comp Neurol* **477**, 412-421, doi:10.1002/cne.20257 (2004).

7 Lang, H. *et al.* Neural stem/progenitor cell properties of glial cells in the adult mouse auditory nerve. *Sci Rep* **5**, 13383, doi:10.1038/srep13383 (2015).

8 Martinez-Monedero, R., Yi, E., Oshima, K., Glowatzki, E. & Edge, A. S. Differentiation of inner ear stem cells to functional sensory neurons. *Dev Neurobiol* **68**, 669-684, doi:10.1002/dneu.20616 (2008).

9 Oshima, K. *et al.* Differential distribution of stem cells in the auditory and vestibular organs of the inner ear. *J Assoc Res Otolaryngol* **8**, 18-31, doi:10.1007/s10162-006-0058-3 (2007).

10 Diensthuber, M. *et al.* Spiral ganglion stem cells can be propagated and differentiated into neurons and glia. *Biores Open Access* **3**, 88-97, doi:10.1089/biores.2014.0016 (2014).

11 Locher, H. *et al.* Distribution and development of peripheral glial cells in the human fetal cochlea. *PLoS One* **9**, e88066, doi:10.1371/journal.pone.0088066 (2014).

12 Kim, Y. Y. *et al.* Hearing loss through apoptosis of the spiral ganglion neurons in apolipoprotein E knockout mice fed with a western diet. *Biochem Biophys Res Commun* **523**, 692-698, doi:10.1016/j.bbrc.2019.12.100 (2020).

13 Pechriggl, E. J. *et al.* Development of the innervation of the human inner ear. *Dev Neurobiol* **75**, 683-702, doi:10.1002/dneu.22242 (2015).

14 Eybalin, M., Norenberg, M. D. & Renard, N. Glutamine synthetase and glutamate metabolism in the guinea pig cochlea. *Hear Res* **101**, 93-101, doi:10.1016/s0378-5955(96)00136-0 (1996).

15 Jung, J. S. *et al.* Semaphorin-5B Controls Spiral Ganglion Neuron Branch Refinement during Development. *J Neurosci* **39**, 6425-6438, doi:10.1523/JNEUROSCI.0113-19.2019 (2019).

Supplementary Table 3

Gene Mm	Prime forward 5'-3'	Prime reverse 5'-3'
Eef1A1	TCCACTTGGTCGCTTTGCT	CTTCTTGTCCACAGCTTTGATGA
Tubb	GCAGTGCGGCAACCAGAT	AGTGGGATCAATGCCATGCT
Actb	CTAAGGCCAACCGTGAAAAGAT	CACAGCCTGGATGGCTACGT
Tubb3	GCCAAGTTCTGGGAGGTCAT	GGGCACATACTTGTGAGAGGA
Prph	CTCTCCAGGATAGCCACAC	GGGCCAAGCTTAGGAATAGG
Isl1	CGACCCAGTCAATGGAAACT	TGGGCTTAGGGTTTTGTTTG
Ntrk2	ACTTCGCCAGCAGTAGCAG	ACCTCAGGGCTGGGGAG

Supplementary table 1. List of qPCR primers used in the study

Supplementary Video Files

The supplementary video 1 shows time lapse imaging of phoenix auditory neuroprogenitors (passage 36) differentiating for 168h. The supplementary videos 2-9 show the impact of glutamate, ATP or kainate (100µM) on $Ca2+$ mobilization by 7 days differentiated auditory neurons from phoenix cells (Sup Video 2-4), human fetus (Sup video 5-6) and spiral ganglion explants of C57Bl6/J mouse pups (P5 postnatal) (Sup Video 7-9) (please refer to the Method section for details). Sup Video 2: phoenix + glutamate; Sup Video 3: phoenix + ATP; Sup Video 4; phoenix + Kainate; Sup Video 5: human auditory neurons + Glutamate; Sup Video 6: human auditory neurons + ATP; Sup Video 7: C57Bl6/J explants + Glutamate; C57Bl6/J explants + ATP; C57Bl6/J explants + Kainate.

Supplementary Methods

Comparison of phoenix transcriptome to publicly available dataset (GSE132925) from Gene Expression Omnibus (GEO).

In order to better characterice our Phoenix cell line, we compared it using public data (GSE132925) from Gene Expression Omnibus (GEO). More specifically, preprocessed normalized count data was used to estimate differentially expressed genes between postnatal day 1 (P1) and postnatal day 30 (P30) in the mouse spiral ganglion neurons using edgeR version 3.28.1 in R. Next, we compared the differentially expressed genes in auditory neuron upon auditory system development and maturation (P1 vs. P30) with our own data (phoenix progenitors vs. differentiated) using hypergeometric probability distribution. We observed significant overlap of differentially expressed genes between the two comparisons (p=0.03420227).
